# Genome-Wide Impact of Folic Acid on DNA Methylation and Gene Expression in Lupus Adipocytes: An In Vitro Study on Obesity

**DOI:** 10.3390/nu17061086

**Published:** 2025-03-20

**Authors:** Leticia L. Souza, Jhulia C. N. L. da Mota, Lucas M. Carvalho, Amanda A. Ribeiro, Cesar A. Caponi, Marcela A. S. Pinhel, Nicolas Costa-Fraga, Angel Diaz-Lagares, Andrea G. Izquierdo, Carla B. Nonino, Ana B. Crujeiras, Carolina F. Nicoletti

**Affiliations:** 1 Applied Physiology and Nutrition Research Group, School of Physical Education and Sport and Faculdade de Medicina, Universidade de Sao Paulo, Sao Paulo 05508-220, Brazil; 2 Center of Lifestyle Medicine, Faculdade de Medicina, Universidade de Sao Paulo, Sao Paulo 05508-220, Brazil; 3 Hospital das Clinicas, Faculdade de Medicina, Universidade de Sao Paulo, Sao Paulo 05508-220, Brazil; 4 Department of Molecular Biology, Sao Jose do Rio Preto Medical School, Sao Jose do Rio Preto 15090-000, Brazil; 5 Department of Health Sciences, Ribeirao Preto Medical School, University of Sao Paulo, Ribeirao Preto 14049-900, Brazil; 6 Epigenomics Unit, Cancer Epigenomics, Translational Medical Oncology Group (ONCOMET), Instituto de Investigacion Sanitaria de Santiago (IDIS), Complejo Hospitalario Universitario de Santiago de Compostela (CHUS/SERGAS), 15706 Santiago de Compostela, Spain; 7 Centro de Investigacion Biomedica en Red Cancer (CIBERONC), ISCIII, 28029 Madrid, Spain; 8 Department of Clinical Analysis, Complejo Hospitalario Universitario de Santiago de Compostela (CHUS/SERGAS), 15706 Santiago de Compostela, Spain; 9 Epigenomics in Endocrinology and Nutrition Group, Epigenomics Unit, Instituto de Investigacion Sanitaria de Santiago de Compostela (IDIS), Complejo Hospitalario Universitario de Santiago de Compostela (CHUS/SERGAS), 15706 Santiago de Compostela, Spain; 10 Centro de Investigacion Biomedica en Red de Fisiopatologia de la Obesidad y Nutricion (CIBEROBN), Instituto de Salud Carlos III, 28029 Madrid, Spain; 11 Rheumatology Division, Hospital das Clinicas, Faculdade de Medicina, Universidade de São Paulo, Sao Paulo 05508-220, Brazil

**Keywords:** in vitro, systemic lupus erythematosus, folic acid, obesity, epigenetics, adipocytes, gene expression, immune modulation

## Abstract

Objective: This in vitro study aimed to investigate the impact of folic acid on DNA methylation and gene expression in adipocytes from subcutaneous adipose tissue of patients with systemic lupus erythematosus (SLE), with a focus on the influence of obesity on these epigenetic changes. Methods: Tissue biopsies were collected from patients with normal weight (NW) and obesity (OBS). Adipocytes were isolated via enzymatic digestion and density separation. Each group was divided into control (standard medium) and folic acid treatment (2 mg/24 h for 48 h) conditions. After treatment, DNA methylation levels were analyzed using the Infinium Methylation EPIC v2.0 Kit, and gene expression analyses were performed by RT-qPCR. A pathway enrichment analysis was conducted using the KEGG database for functional insight. Results: Folic acid induced differential methylation at 755 CpG sites in NW adipocytes, which were associated with immune regulation, including MAPK signaling. Also, OBS adipocytes showed methylation changes at 92 CpG sites, affecting pathways related to metabolic regulation, such as cAMP signaling. *LEP* gene expression was upregulated (5.2-fold) in OBS adipocytes, while *CREM2* expression was increased (2.8-fold) in NW adipocytes after treatment. These gene expression differences underscore weight-dependent responses to folic acid, with *LEP* upregulation in OBS cells suggesting links to metabolic dysregulation and *CREM2* upregulation in NW cells potentially contributing to immune modulation. Conclusions: Folic acid treatment exerts distinct epigenetic and gene expression effects in adipocytes of SLE patients, modulated by obesity status. This weight-dependent response, marked by changes in pathways relevant to immune and metabolic function, highlights the need for further investigation into how nutrient-based interventions might support SLE management. From a clinical perspective, this study underscores the potential of targeted nutrient-based interventions to address immunometabolic dysfunctions in SLE patients. Further research could explore folic acid supplementation as a complementary approach to personalized treatment strategies, particularly for patients with obesity.

## 1. Introduction

Systemic lupus erythematosus (SLE) is a rheumatic disease characterized by the loss of immune system homeostasis, primarily driven by the production of autoantibodies [1]. SLE presents various systemic manifestations mediated by pro-inflammatory cytokines, which contribute to a significant decline in patients’ quality of life [1,2]. Beyond musculoskeletal involvement and joint manifestations, SLE may affect the skin, cardiovascular, pulmonary, renal, and gastrointestinal systems, underscoring the complexity and heterogeneity of the disorder [3]. Notably, SLE predominantly affects women (90%), with a global prevalence of approximately 3.4 million people [4].

It is known that SLE can be associated with other pathologies, including arthritis [5], atherosclerosis [6], nephritis [7], and metabolic disorders such as diabetes mellitus [8], metabolic syndrome [9], and obesity [10].

Global obesity parameter highlights a growing public health concern, with data indicating that approximately 2.5 billion adults aged 18 and older are considered overweight, and around 890 million live with obesity, according to the World Health Organization (WHO) [11]. In this context, obesity prevalence among patients with SLE has gained significant attention in recent years [8,12,13] due to its potential role in the immunometabolic axis in SLE prognosis [12]. Obesity is characterized as a chronic inflammatory condition marked by excessive adipose tissue accumulation and the dysregulated production of adipokines and pro-inflammatory cytokines [14]. When addressing the SLE–obesity axis, these data suggest that excess weight and obesity may be associated with increased disease activity, worse prognosis, and reduced treatment response [12,15].

Emerging evidence suggests that epigenetic regulation plays a crucial role in both SLE and obesity, influencing immune responses, inflammation, and metabolic dysfunction [16]. DNA methylation, one of the most well-characterized epigenetic mechanisms, has been shown to differ significantly between individuals with obesity and those with normal weight, particularly in subcutaneous adipose tissue (SAT), where aberrant methylation is linked to adipocyte dysfunction and systemic inflammation [17]. These alterations may contribute to the chronic inflammatory state observed in obesity, potentially exacerbating autoimmune conditions like SLE. Given that both diseases share inflammatory pathways and epigenetic modifications, understanding their interplay may provide insights into disease mechanisms and potential therapeutic strategies.

A range of factors contribute to the etiopathogenesis of autoimmune diseases, with epigenetics emerging as a key area of focus [16,18]. Epigenetics encompasses heritable modifications in gene activity that do not involve changes to the primary nucleotide sequence [19]. These modifications, including DNA methylation, histone modifications, and regulation by non-coding RNAs, influence gene expression patterns [20]. DNA methylation, mediated by DNA methyltransferases (DNMTs), is one of the most common epigenetic mechanisms in gene regulation, promoting gene silencing by inhibiting transcription [19].

In SLE, numerous studies have identified altered DNA methylation patterns as key contributors to the development of autoimmunity and the loss of self-tolerance [21,22,23,24]. Specifically, global and gene-specific hypomethylation has been observed, particularly in genes associated with inflammatory processes. Pro-inflammatory cytokines such as IL-6 [25], IL-10, IL-13 [26], and IL-17A [27,28] are regulated through epigenetic remodeling and show hypomethylation in SLE, leading to their elevated expression in CD4+ T cells. This upregulation contributes to tissue damage and autoantibody production, further driving disease pathology.

Dietary nutrients can modulate epigenetic mechanisms by influencing DNMT activity or altering substrate availability. One-carbon metabolism plays a central role in these processes, as it provides methyl groups from S-adenosyl-methionine (SAM) to DNMTs for DNA methylation. Key micronutrients, such as folic acid, are essential cofactors in this pathway and may influence DNA methylation patterns and gene expression [29]. The relationship between methyl-donor nutrients and DNA methylation has gained increasing attention, given their potential implications for disease modulation [20,30,31,32].

Epigenetic modifications are dynamic, allowing cells and tissues to differentiate and adapt in response to environmental factors, making them attractive targets for therapeutic intervention. Studies have begun to explore the impact of folate supplementation on DNA methylation patterns, with evidence from in vitro studies, animal models, and human trials suggesting that methyl-donor nutrients can modify DNA methylation levels [20,30,33]. However, research on the effects of folate supplementation in SLE remains limited and inconclusive. In lupus-prone mouse models, folate immunotherapy was shown to reduce SLE-related symptoms and improve survival [34]. Additionally, a diet rich in folic acid was associated with increased DNA methylation levels, particularly in B lymphocytes of autoimmune mice, suggesting a potential immunomodulatory role [32].

Despite these findings, the impact of folic acid supplementation on obesity-related epigenetic modifications in the context of SLE remains largely unexplored. The primary objective of this study was to evaluate whether folic acid treatment modulates DNA methylation profiles and gene expression in adipocytes isolated from patients with systemic lupus erythematosus (SLE). The secondary objective was to investigate whether these epigenetic and transcriptional changes differ according to obesity status.

## 2. Methods

### 2.1. Ethical Considerations

This observational and experimental in vitro study was approved by the local Ethical Committee (Commission for Analysis of Research Projects, CAPPesq; approval: 47317521.8.0000.0068), and patients provided informed consent before participating. The procedures were conducted in accordance with the Declaration of Helsinki revised in 2008. As a primary endpoint, this clinical trial assessed the DNA methylation profile in adipose tissue. As secondary endpoints, the study included gene expression analyses. This study was conducted following the STROBE (Strengthening the Reporting of Observational Studies in Epidemiology) guidelines [35].

### 2.2. Patients with SLE Donors of Subcutaneous Adipose Tissue

Two female patients with a confirmed diagnosis of SLE were recruited from the Lupus Outpatient Clinic of the Rheumatology Division at Hospital das Clínicas (HCFMUSP), Faculdade de Medicina, Universidade de São Paulo, São Paulo, Brazil, in April 2023. The study included one patient with normal weight (NW) and one patient with obesity (OBS) (Table 1). Inclusion criteria were as follows: pre-menopausal status; aged between 18 and 45 years; inactive disease; prednisone use ≤ 10 mg/day and hydroxychloroquine use at a stable dose. Additionally, exclusion criteria included chronic disease (diabetes mellitus, arterial hypertension, cancer), current smokers, current anticoagulants and/or methotrexate use, current infection, pregnancy, and current use of any supplementation with methyl-donor micronutrients (e.g., vitamin B12, folic acid).

### 2.3. Experiment Design: Cell Culture

Subcutaneous adipose tissue (SAT) biopsies were collected from the abdominal region of patients with NW and OBS. Adipocytes were isolated following the Martin Rodbell method from 1964, with adaptations by Peres and Curi in 2005. This method involves dissociating the extracellular matrix and isolating adipocytes based on density [36]. The SAT samples were dissected into small, uniform pieces with scalpel blades and placed into 50 mL conical tubes containing a collagenase solution (1 mg/mL, Type I Collagenase, Gibco™—Thermo Fisher Scientific, Waltham, MA, USA) in a culture medium composed of Dulbecco’s Modified Eagle Medium (DMEM) with glutamine, pyruvate, glucose, and NaHCO_3_, supplemented with HEPES, bovine serum albumin, ultrapure water, and penicillin-streptomycin (10,000 IU/mL—Thermo Fisher Scientific, Waltham, MA, USA). DMEM has been widely used in adipocyte cultures due to its nutrient composition, which supports adipocyte viability and function ex vivo. The medium and supplements were refreshed every 24 h for 48 h to maintain optimal culture conditions. The samples were incubated in a water bath at 37 °C for approximately 30 min with periodic agitation.

Following incubation, the samples were centrifuged at 400× *g* for 30 s at room temperature. The supernatant containing adipocytes was carefully aspirated, washed twice with the culture medium, and adjusted to pH 7.4. Adipocyte counts were conducted using a Neubauer chamber, and cell viability was assessed with Trypan Blue dye (Gibco™, Thermo Fisher Scientific, Waltham, MA, USA).

**Figure 1:**
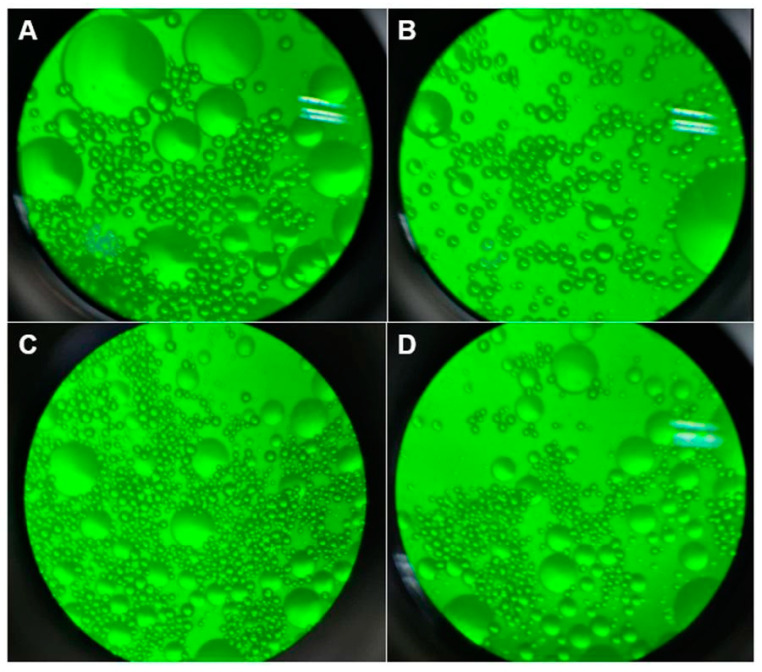
SLE adipocyte cell culture. (**A**) Obesity control group. (**B**) Obesity treatment group. (**C**) Normal weight control group. (**D**) Normal weight treatment group.

A total of 1,600,000 adipocytes were obtained at a concentration of 400,000 cells/mL in the OBS group, while 613,333 adipocytes were obtained at 153,333 cells/mL in the NW group. Equal numbers of cells were distributed into 40 mL cell culture flasks labeled according to group (NW or OBS) and intervention (control or folic acid treatment): (1) NW control, (2) NW treatment, (3) OBS control, and (4) OBS treatment. After seeding, all flasks were supplemented with 4 mL of culture medium. Flasks designated for folic acid treatment received an additional 1 mL of folic acid (2 mg/flask) dissolved in NaOH (0.02 g NaOH). The NaOH concentration was minimal and diluted upon supplementation, with pH adjustments ensuring compatibility with adipocyte culture. However, we acknowledge that the inclusion of a vehicle control (NaOH alone) would have strengthened the specificity of the treatment effects (Figure 1).

Nucleic acids were extracted after the 48 h incubation. RNA and DNA extractions were performed using the RNeasy™ Mini Kit (Qiagen, Hilden, Germany) and QIAamp™ DNA Mini Kit (Qiagen, Hilden, Germany), respectively. The contents of each flask were homogenized for 30 s, transferred to 15 mL conical tubes, and centrifuged at 400× *g* for 30 s. The supernatant was divided equally for RNA and DNA extraction, with procedures following the manufacturer’s protocols. Nucleic acid quantification was carried out using a NanoVue™ Spectrophotometer (Cytiva, formerly GE Healthcare, Chicago, IL, USA), with purity assessed by 260 nm/280 nm absorbance ratios.

### 2.4. DNA Methylation Analysis

Extracted DNA samples were treated with sodium bisulfite using the EZ DNA Methylation-Gold Kit (Zymo Research, Irvine, CA, USA) as per manufacturer instructions. DNA methylation profiling was conducted using the Infinium Methylation EPIC v2.0 Kit (Illumina™, San Diego, CA, USA), following Illumina’s Methylation Protocol Guide (2015). Approximately 100 ng of bisulfite-treated DNA underwent denaturation, neutralization, and isothermal amplification. This product was enzymatically fragmented, precipitated, and resuspended in a buffer solution, then applied to BeadChip slides for hybridization. After incubation at 48 °C for 16 h, slides were washed, and unhybridized fragments were removed before undergoing base extension and amplification. The detection of methylated cytosines was performed by scanning with the Illumina iScanSQ™ system.

Raw methylation data (IDAT files) were processed within the R statistical environment (v4.4.0), utilizing Bioconductor packages for high-throughput genomic data analysis. Preprocessing and quality control were performed using the R package minfi (version: 1.52.1). This included background correction, normalization (functional normalization), and filtering of low-quality probes (detection *p*-value > 0.01) and cross-reactive probes. Beta values, representing methylation levels, were calculated as β = M/(M + U + 100), where M and U denote methylated and unmethylated intensities, respectively. Differential methylation analysis between groups was conducted using the limma package (version: 3.62.2), which employs linear models to identify differentially methylated CpGs with adjusted *p*-values < 0.05 and absolute methylation differences (Δβ) greater than ±0.3. A pathway enrichment analysis was conducted using modEnrichr, with KEGG pathways considered significant at *p*-value < 0.05

### 2.5. Gene Expression Analysis by RT-qPCR

Reverse transcription was performed using the High-Capacity cDNA Reverse Transcription Kit (Applied Biosystems, Foster City, CA, USA) in an MJ Research PTC-100^®^ thermocycler. DNA methyltransferase 1 (*DNMT1*), interleukin 6 (*IL-6*), CAMP responsive element modulator (*CREM2*), leptin (*LEP*), and adiponectin (*ADIPOQ*) expression was quantified by RT-qPCR on a Step One Plus Real-Time PCR System (Applied Biosystems, Thermo Fisher Scientific, Waltham, MA, USA) using TaqMan™ MGB 6-FAM probes (Applied Biosystems, Thermo Fisher Scientific, Waltham, MA, USA) and Gene Expression Master Mix (Applied Biosystems, Thermo Fisher Scientific, Waltham, MA, USA). Relative gene expression was calculated using the 2^−ΔΔCt^ method, with GAPDH and β-actin (ACTB) as endogenous controls. A 1.5-fold change in expression was set as the threshold for significant differential expression [37].

## 3. Results

### 3.1. Folic Acid Effects on DNA Methylation Profile

**Figure 2:**
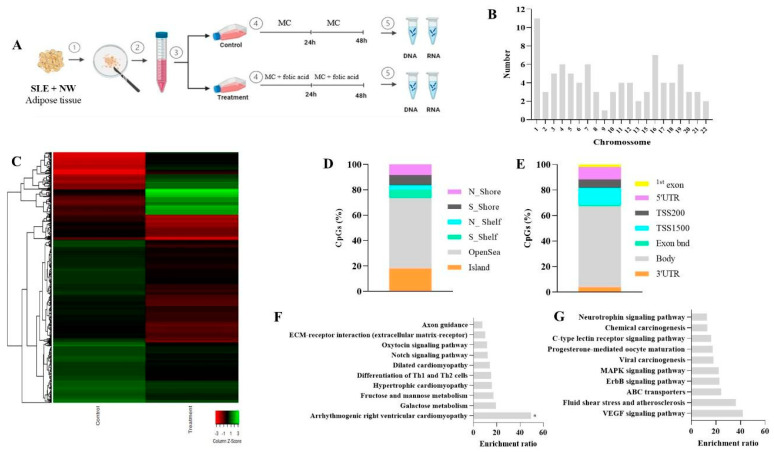
Genomic features of differently methylated CpG sites in normal weight group (control vs. treatment). (**A**) Experimental cell culture design. (**B**) Distribution of different CpG sites across autosomes chromosomes. (**C**) Heat maps of methylation differences discriminating control and treated SLE adipocytes analyzed on the Infinium HumanMethylation450 BeadChip platform. DNA methylation values were represented as colors, with red indicating more DNA methylation and green less DNA methylation. The row represents individual CpGs, and the column represents groups. (**D**) CpG context of the identified differently methylated CpG sites. (**E**) Genomic localization of the identified differently methylated CpG sites. (**F**) Significant pathway enrichment results using the identified hypermethylated CpG sites. (**G**) Significant pathway enrichment results using the identified hypomethylated CpG sites. SLE: systemic lupus erythematous. NW: normal weight. MC: medium culture. *: adjusted *p* < 0.05.

In the NW cell cultures, 755 CpG sites across 537 unique genes were found to be differentially methylated between control and folic acid-treated cultures. Of these, 188 CpGs were hypermethylated, while 567 were hypomethylated following treatment. The majority of these differentially methylated CpG sites (55.4%) were in OpenSea regions, with 18.1% in CpG islands. Distribution of these sites also varied within the gene structure: 63.5% were situated within the gene body, and 31.5% in the promoter region. Enrichment analysis of genes with hypermethylated CpGs (158 genes) and hypomethylated CpGs (55 genes) revealed significant associations with MAPK-related pathways, highlighting potential regulatory roles of folic acid in signaling pathways related to immune and inflammatory responses (Figure 2).

**Figure 3:**
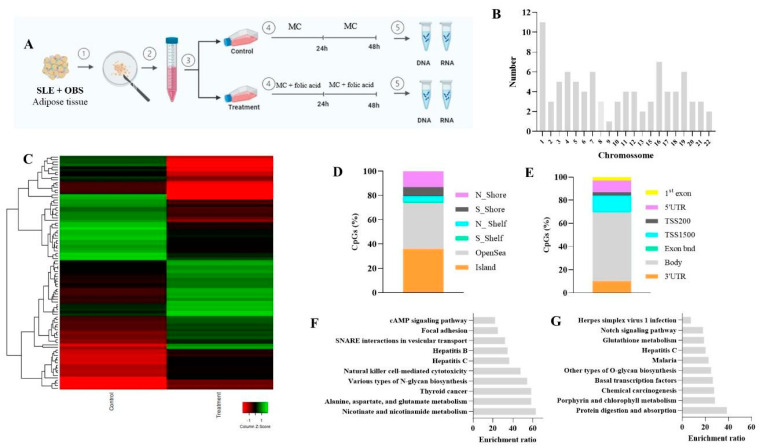
Genomic features of differently methylated CpG sites in obesity group (control vs. treatment). (**A**) Experimental cell culture design. (**B**) Distribution of differently CpG sites across autosomes chromosomes. (**C**) Heat maps of methylation differences discriminating control and treated SLE adipocytes analyzed on the Infinium HumanMethylation450 BeadChip platform. DNA methylation values were represented as colors, with red indicating more DNA methylation and green less DNA methylation. The row represents individual CpGs, and the column represents groups. (**D**) CpG context of the identified differently methylated CpG sites. (**E**) Genomic localization of the identified differently methylated CpG sites. (**F**) Significant pathway enrichment results using the identified hypermethylated CpG sites. (**G**) Significant pathway enrichment results using the identified hypomethylated CpG sites. SLE: systemic lupus erythematous. OBS: obesity. MC: medium culture.

In contrast, in OBS cell cultures, 92 CpG sites across 71 unique genes were differentially methylated between control and treatment conditions. Of these, 51 CpG sites were hypermethylated, and 41 were hypomethylated in the treated culture. A distribution analysis indicated that 38% of these CpG sites were in OpenSea regions and 35.9% in CpG islands, with 59.4% found within the gene body and 25% in promoter regions. Enrichment analysis of 15 hypermethylated and 24 hypomethylated genes revealed associations with cAMP-related pathways in the hypomethylated profile, suggesting that folic acid may influence metabolic regulation pathways specific to obesity-related contexts (Figure 3).

**Figure 4:**
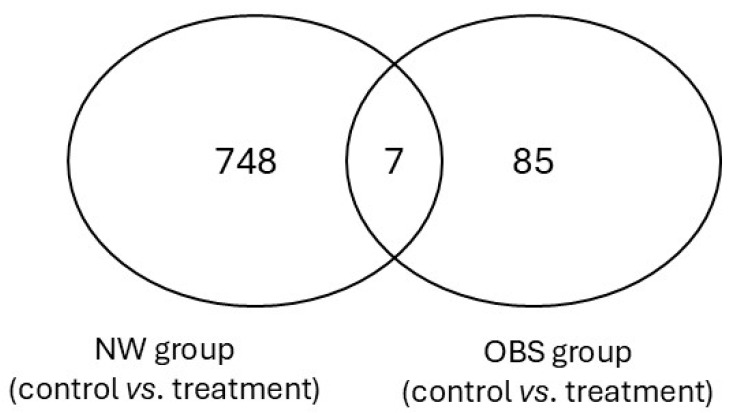
Venn diagram depicting overlaps of differentially methylated CpG sites identified when control culture was compared to treated culture in normal weight and obesity groups. Seven CpG sites were consistently differentially methylated in SLE normal weight and obese adipocytes.

Interestingly, when comparing differentially methylated CpG sites across NW and OBS cell cultures, only seven CpG regions overlapped between the groups (Figure 4). Table 2 provides detailed information on the gene regions and associated functions of these overlapping CpG sites. This minimal overlap may reflect distinct epigenetic responses to folic acid depending on weight status, which could contribute to understanding the influence of folic acid in obesity- and SLE-specific epigenetic landscapes.

### 3.2. Folic Acid Effects on DNMT1, IL-6, CREM2, LEP, and ADIPOQ Expression

**Figure 5:**
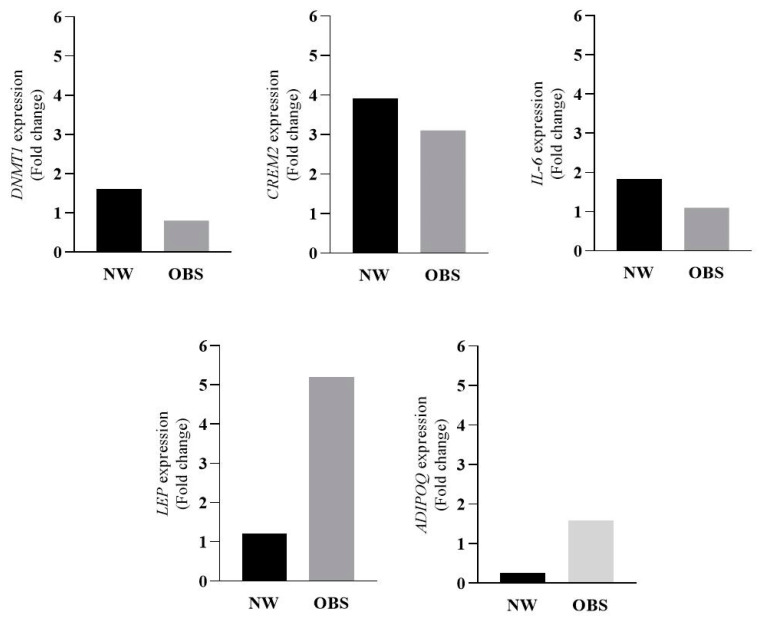
Effects of folic acid treatment on *DNMT1*, *IL-6*, *CREM2*, *LEP*, and *ADIPOQ* expression in cell culture of study groups (data are presented as mean). NW: normal weight; OBS: obesity.

Gene expression analyses showed no significant differences in *IL-6*, *ADIPOQ*, or *DNMT1* expression levels between control and folic acid-treated cultures for both NW and OBS groups, based on the 2^−ΔΔCt^ calculation. However, notable gene expression changes were observed for *LEP* and *CREM2*. Specifically, *LEP* expression was upregulated by 5.2-fold in the OBS treatment culture, suggesting potential modulatory effects of folic acid on obesity-related adipocyte function. Conversely, *CREM2* was upregulated by 2.8-fold in the NW treatment culture, highlighting a possible connection between folic acid and cAMP-related signaling pathways in non-obese conditions (Figure 5).

## 4. Discussion

To our knowledge, this study represents the first in vitro examination of folic acid’s impact on DNA methylation and gene expression profiles in adipocytes from patients with SLE and obesity contexts. Our results indicate that folic acid exerts weight-dependent epigenetic effects, altering methylation across distinct CpG sites in NW versus OBS adipocytes, with notable changes in the expression of *LEP* and *CREM2* genes. These findings highlight a nuanced relationship between folic acid treatment, baseline weight status, and potential regulatory mechanisms relevant to SLE pathology.

The substantial difference in CpG methylation profiles between NW and OBS cultures suggests that obesity influences the epigenetic responsiveness to folic acid, potentially altering key regulatory pathways involved in immune and metabolic homeostasis. NW adipocytes exhibited a greater number of differentially methylated CpGs compared to OBS cultures, with a predominance of hypermethylation in genes associated with the MAPK pathway. This pathway plays a critical role in immune signaling and autoimmune regulation and is strongly implicated in SLE pathogenesis, where excessive MAPK activity contributes to pro-inflammatory cytokine expression and immune cell dysfunction [37]. The hypermethylation of genes in this pathway suggests potential transcriptional repression, which could have therapeutic implications, as MAPK inhibitors are being explored as potential modulators of autoimmune responses [38,39]. Our findings indicate that folic acid may exert a differential regulatory effect on MAPK activity, potentially attenuating pro-inflammatory signaling in non-obese conditions.

In contrast, the predominant hypomethylation in OBS adipocytes revealed enrichment in the cAMP signaling pathway, a mechanism that directly interacts with CREM, a key transcriptional regulator in SLE pathogenesis [40]. Aberrant *CREM* activation in SLE lymphocytes has been associated with reduced IL-2 production, T-cell anergy, and heightened inflammatory responses [41,42]. The observed hypomethylation in this pathway suggests a potential increase in *CREM* expression, possibly amplifying immune and metabolic dysfunctions in obese adipocytes, highlighting a weight-dependent effect of folic acid supplementation.

These findings underscore the complex interplay between nutrition, epigenetics, and molecular signaling pathways in obesity and SLE, suggesting that folic acid supplementation may modulate inflammatory responses differently depending on baseline metabolic status. The differential influence on MAPK and CREM/cAMP pathways suggests potentially opposing mechanisms, where MAPK repression may be beneficial in reducing inflammation in non-obese individuals, while CREM/cAMP activation could exacerbate pathological processes in obese adipocytes. These effects highlight the need to consider individual metabolic and epigenetic contexts when evaluating the impact of folic acid on immunometabolic regulation in autoimmune diseases.

A critical aspect to consider is the limited overlap of only seven CpG sites between NW and OBS cultures, pointing to a set of universally responsive sites to folic acid treatment. These CpG sites, though few, may represent core epigenetic mechanisms relevant to SLE and obesity and could serve as potential therapeutic targets. Their functions—spanning immune modulation, neuroprotection, and extracellular matrix regulation—suggest that even a limited overlap in CpG methylation can hold significant relevance for understanding folic acid’s role in disease modulation. Further investigation into these core CpG sites in clinical populations could clarify their potential as biomarkers or targets for personalized treatment approaches in SLE and obesity.

The upregulation of *LEP* in OBS cultures and *CREM2* in NW cultures adds a layer of functional significance to these findings. LEP plays a critical role in adipose tissue homeostasis, energy balance, and immune regulation, and its dysregulation has been linked to metabolic disturbances in obesity and inflammatory states, including SLE [43,44]. The observed LEP upregulation in the OBS treatment group aligns with evidence suggesting that folic acid influences lipid metabolism, insulin sensitivity, and inflammatory pathways in obesity models [43,44]. Given that leptin resistance is a hallmark of obesity and has been associated with immune dysfunction in SLE, this increase may reflect an adaptive response or, conversely, a potential exacerbation of metabolic dysregulation and chronic inflammation in these patients. Further studies are needed to determine whether this modulation of LEP by folic acid translates into beneficial or detrimental metabolic and immune outcomes in SLE-affected individuals.

Conversely, CREM2 upregulation in NW cultures may indicate a distinct, cAMP-mediated response to folic acid in non-obese conditions. CREM2 is involved in the regulation of immune function, particularly in T-cell activation and cytokine signaling, which are relevant to SLE pathogenesis [45]. The differential response observed between NW and OBS groups suggests that baseline metabolic status may shape the epigenetic and transcriptional effects of folic acid, potentially influencing immune modulation in opposing directions. While *CREM2* upregulation in NW adipocytes could enhance anti-inflammatory pathways, it might also exacerbate certain immune alterations characteristic of SLE, warranting further investigation into the precise downstream effects of this regulation.

However, it is important to note that the observed lack of change in *DNMT1*, *IL-6*, and *ADIPOQ* expression across conditions suggests that folic acid’s effects are highly gene- and pathway-specific. This specificity underscores the complexity of folic acid’s interaction with DNA methylation and gene expression. Furthermore, the present study’s findings should be interpreted with caution due to the short intervention period (48 h), which may not fully capture the extent of gene expression changes induced by folic acid. Future studies could benefit from prolonged exposure to better understand the durability of these epigenetic changes.

### 4.1. Limitations

A limitation of this study is the heterogeneity in folic acid dosing and treatment duration found in the literature, which complicates direct comparisons. Longer treatment times and varied doses could provide a more comprehensive picture of folic acid’s modulatory potential on methylation and gene expression. The treatment duration of 48 h may not be sufficient to capture the long-term effects of folic acid on DNA methylation and gene expression. Chronic effects may differ significantly from short-term responses. Additionally, future studies should aim to replicate these findings in primary human adipocytes and explore the broader implications of *LEP* and *CREM2* expression changes in clinical SLE populations. Expanding upon these results, in vivo models or patient cohorts could further elucidate folic acid’s impact on inflammatory and metabolic pathways in SLE, particularly in individuals with obesity, thereby informing more targeted epigenetic therapies. Furthermore, the findings obtained in this study suggest a broader approach to more accurately analyzing the use of folic acid supplementation as one of the possibilities in personalized therapy for SLE, particularly in the presence of obesity. In this sense, there is a need for rigorous studies, such as randomized controlled trials (RCTs), involving adequately sized populations to validate these findings [46,47,48].

### 4.2. Perspectives for Clinical Practice

Applying the in vitro results from this study to clinical practice presents a challenge, as cell culture studies do not fully replicate the complexity of in vivo systems. However, the findings from this research can serve as a foundation for developing therapeutic strategies and predictive models that could eventually be incorporated into clinical practice. Artificial intelligence (AI) can play a crucial role in translating these findings, especially in the areas of personalized medicine and predictive outcomes. Patient follow-up on folic acid treatment could involve the continuous monitoring of DNA methylation and gene expression through technologies such as genetic sequencing and biomarker testing.

With the DNA methylation data and observed responses in cell cultures, AI could be used to develop algorithms to help personalize treatments for patients with SLE and obesity. For example, algorithms could suggest the optimal folic acid dosage or other treatments based on the patient’s epigenetic profile. Additionally, by collecting clinical and epigenetic response data over time, AI could be used to adjust therapy, taking into account the patient’s baseline epigenetic and metabolic status, ensuring the most effective treatment over time.

## 5. Conclusions

This study provides new insights into the weight-specific epigenetic effects of folic acid treatment in adipocyte cultures within the context of SLE. The differential methylation and gene expression patterns observed in NW versus OBS cultures reveal that folic acid has distinct regulatory effects on inflammation and metabolism-related pathways. Notably, folic acid treatment was associated with MAPK pathway hypermethylation in NW cultures and cAMP pathway hypomethylation in OBS cultures, suggesting that the baseline weight status may modulate the epigenetic impact of folic acid on key cellular processes relevant to SLE pathology and metabolic health. The specific upregulation of *LEP* in OBS and *CREM2* in NW cultures further highlights the complex and context-dependent nature of folic acid’s influence on gene expression, indicating potential therapeutic pathways for tailored folic acid interventions in SLE, particularly in individuals with obesity.

These findings contribute to a growing understanding of how micronutrient interventions can impact disease-relevant epigenetic landscapes in an individualized manner. However, future longitudinal studies and clinical trials and across extended intervention periods is warranted to validate these effects and explore their therapeutic implications. By illuminating the interplay between folic acid, DNA methylation, and weight status, this study paves the way for more personalized approaches to managing SLE and potentially other autoimmune conditions through epigenetic modulation.

## Figures and Tables

**Table 1:** Clinical characteristics of lupus patients participating in the study.

	NW Patient	OBS Patient
Age (years)	37	34
SLEDAI-2K	0	0
Hydroxychloroquine use (mg/day)	400	400
Associated comorbidities	-	-
BMI (kg/m^2^)	21.0	31.1
Fat mass (%)	28.7	38.4

NW: normal weight; OBS: obesity; SLEDAI: Systemic Lupus Erythematosus Disease Activity Index 2000, BMI: body mass index.

**Table 2:** Comparison of 7 overlapping CpG regions among control and treatment cell cultures from normal weight and obesity groups.

CpG	Gene (Localization)	Function
cg00912518	*MBP* (body)	Encodes myelin proteins essential for the myelin sheath in the nervous system, with variants also acting in bone marrow and the immune system.
cg07342845	*TAF4* (body)	Encodes a transcription factor subunit for transcription initiation and modulation of the response to regulatory signals, with implications in neurodegenerative diseases.
cg20478468	*EIF4E* (TSS1500)	Part of the translation initiation factor complex 4F to start translation. Acts as a proto-oncogene, with its expression and activation associated with tumorigenesis.
cg03689146	Unknown (island)	-
cg18605377	Unknown (island)	-
cg26669806	*COMP* (body)	Non-collagenous extracellular matrix protein. Structural functions.
cg15319704	*KLF5* (TSS1500; 5’UTR)	Involved in the promotion and suppression of cell proliferation.

*MBP*: Myelin Basic Protein. *TAF4*: TATA-box Binding Protein Associated Factor 4. *EIF4E*: Eukaryotic Translation Initiation Factor 4E. *COMP*: Cartilage Oligomeric Matrix Protein. *KLF5*: Krüppel-like Factor 5 Transcription Factor.

## Data Availability

The datasets generated during and/or analyzed during this study are available from the corresponding author on reasonable request due to the proprietary nature of the data.
